# Clinical Approach to Euvolemic Hyponatremia

**DOI:** 10.7759/cureus.35574

**Published:** 2023-02-28

**Authors:** Pramod Reddy

**Affiliations:** 1 Internal Medicine, University of Florida College of Medicine - Jacksonville, Jacksonville, USA

**Keywords:** siadh, reset osmostat, psychigenic polydipsia, cerebral salt wasting, acute hyponatremia

## Abstract

Euvolemic hyponatremia is frequently encountered in hospitalized patients and the syndrome of inappropriate antidiuretic hormone secretion (SIADH) is the most common cause in most patients. SIADH diagnosis is confirmed by decreased serum osmolality, inappropriately elevated urine osmolality (>100 mosmol/L), and elevated urine sodium (Na) levels. Patients should be screened for thiazide use and adrenal or thyroid dysfunction should be ruled out before making a diagnosis of SIADH. Clinical mimics of SIADH like cerebral salt wasting and reset osmostat should be considered in some patients. The distinction between acute (<48 hours) versus chronic (>48 hours or without baseline labs) hyponatremia and clinical symptomatology are important to initiate proper therapy. Acute hyponatremia is a medical emergency and osmotic demyelination syndrome (ODS) occurs commonly when rapidly correcting any chronic hyponatremia. Hypertonic (3%) saline should be used in patients with significant neurologic symptoms and maximal correction of serum Na level should be limited to <8 mEq over 24 hours to prevent the ODS. Simultaneous administration of parenteral desmopressin is one of the best ways to prevent overly rapid Na correction in high-risk patients. Free water restriction combined with increased solute intake (e.g., urea) is the most effective therapy to treat patients with SIADH. 0.9% saline acts as a hypertonic solution in patients with hyponatremia and should be avoided in the treatment of SIADH due to rapid fluctuations in serum Na levels. Dual effects of 0.9% saline resulting in rapid correction of serum Na during infusion (inducing ODS) and post-infusion worsening of serum Na levels are described in the article with clinical examples.

## Introduction and background

Sodium (Na) and water excretion in the urine are regulated independently in the human body. Sodium levels are regulated by aldosterone, natriuretic peptides, and the antidiuretic hormone (ADH) which regulates water excretion in response to changes in water intake. Normal serum sodium levels are in the range of 135-145 mEq/L and normal urine osmolality ranges between 40-1200 mosmol/L. Urine osmolality provides an indirect measure of the activity of ADH. Hyponatremia is defined as serum sodium (Na) concentration below 135 mEq/L and most hyponatremic patients have an associated low "measured" serum osmolality (normal range 275-295 mosmol/kg), which is termed hypotonic hyponatremia. Patients with hyponatremia usually have a low “calculated” serum osmolality (2 x Na in mEq/L or mosmol/L) + (glucose in mg/dL ÷ 18) + (blood urea nitrogen (BUN) in mg/dL ÷ 2.8), due to the predominant contribution from serum sodium. Physicians should obtain a “measured” serum osmolality in evaluating any form of hyponatremia and it is important to obtain it when intravenous (IV) fluids are not running, which can significantly alter the measured serum osmolality.

Symptoms of hyponatremia

Symptoms of hyponatremia are often nonspecific (concentrated urine, headache, nausea, vomiting, restlessness, confusion, depressed mood, psychosis, hallucinations, lack of attention, impaired memory, muscle cramps, and tremor) and even the smallest reduction in serum Na in elderly patients can result in cognitive decline and increased falls. Patients with moderate to severe neurologic symptoms (seizures, severe somnolence, dysarthria, and hemiplegia) require urgent treatment to prevent brain herniation and death.

Brain adaptation to hyponatremia

Within minutes after the drop in serum Na, brain astrocytes begin to lose intracellular solutes (potassium and osmolytes). This brain adaptation to hypotonicity is usually complete within two days and while it prevents the development of brain edema, it also makes the patients prone to develop osmotic demyelination syndrome (ODS) after rapid correction of serum Na [1]. Hyponatremia should be considered chronic after 48 hours of onset or when no baseline labs are available [2]. ODS is rare in patients with acute hyponatremia that has developed over the past few hours since cerebral adaptation is in the beginning stages. The clinical manifestations of ODS are typically delayed for two to six days before manifesting with bulbar symptoms (dysarthria, dysphagia, brisk jaw jerk), psychiatric disturbances, catatonia, dystonia, tremors, myoclonic jerks, limb paralysis, locked-in state, seizures, lethargy, coma, and death. Symptoms in ODS patients are frequently irreversible, and a 40% functional impairment was noted in a Swedish study [3]. The vast majority of the reported cases of ODS occur in patients admitted with a serum Na of <120 mEq/L, likely reflecting the aggressive administration of saline leading to a rapid increase in the serum Na level during infusion. Patients with certain comorbid conditions (malnutrition, alcoholism, severe liver disease, and hypokalemia) are prone to develop ODS with serum Na levels in the range of 120-125 mEq/L.

## Review

The initial diagnostic approach to a patient with hyponatremia consists of a thorough medical history (e.g., amount of water intake and medication use) and recent surgical history (e.g., prostate or intrauterine procedures). Volume status should be assessed by a focused physical examination to rule out edema (hypervolemia) and orthostasis (hypovolemia). In euvolemic patients, selected laboratory tests (e.g., measured serum osmolality, serum glucose, lipid profile, and total protein albumin gap) will help to rapidly rule out hypertonic or isotonic hyponatremia and the remaining patients are likely to have hypotonic hyponatremia.

The syndrome of inappropriate ADH secretion (SIADH) is the most common cause of euvolemic hypotonic hyponatremia (see Table 1 for etiology).

**Table 1:** Etiology of SIADH ADH: antidiuretic hormone; MDMA: methylenedioxymethamphetamine; SIADH: syndrome of inappropriate antidiuretic hormone secretion

Medical Conditions	Drugs
Acute illness, emotional stress, psychosis, pain, and nausea	Antidepressants & antipsychotics: Selective serotonin reuptake inhibitors, tricyclics, venlafaxine, phenothiazines, butyrophenones, monoamine oxidase inhibitors
Exercise-associated hyponatremia	
Pulmonary diseases: Any form of pneumonia, acute respiratory failure, pneumothorax, and acute respiratory distress syndrome	Anticonvulsants: Carbamazepine, sodium valproate, lamotrigine
HIV infection, adrenal insufficiency, hypothyroidism	
Central nervous system (CNS) disorders: Stroke, hemorrhage, infection, trauma, and psychosis	Anticancer drugs: Vinca alkaloids, ifosfamide, melphalan, cyclophosphamide, methotrexate
Surgical procedures: Orthopedic surgeries (e.g., hip & knee replacement) and trans-sphenoidal pituitary surgery	Vasopressin analogues: Desmopressin, oxytocin, terlipressin, vasopressin
Ectopic production of ADH: Most often due to small cell carcinoma of the lung (extrapulmonary small cell carcinoma in a few cases). Other causes include head and neck cancer, olfactory neuroblastoma, C-cell carcinomas of the thyroid, and pancreatic cancer	Miscellaneous: Opiates, nonsteroidal anti-inflammatory drugs, MDMA (ecstasy), levamisole, clofibrate, interferon, amiodarone, bromocriptine, angiotensin-converting enzyme inhibitors
Hereditary and idiopathic causes	

Inappropriate versus appropriate release of ADH

The normal osmostat for vasopressin release is fixed between 275 and 295 mosmol/kg and hyponatremia with resulting hypotonicity causes swelling of cerebral osmoreceptor cells which normally suppresses the ADH release from the posterior pituitary gland. Low ADH levels are indirectly manifested by very low urine osmolality (around 50 mosmol/kg) promoting the excretion of excess body water in dilute urine. However, in patients with SIADH, there is an inability to suppress the secretion of ADH from the negative feedback and persistently elevated ADH levels impair the water excretion by the kidneys thereby producing concentrated urine and elevated urine osmolality. Since serum ADH levels are not readily available, for all practical purposes any euvolemic hyponatremia patient with a urine osmolality greater than 100 mosmol/kg (twice the lowest osmolality achievable by the kidneys) is assumed to have an “inappropriate” release of ADH. An increase in total body water (TBW) lowers the serum Na concentration and tonicity by dilution and also affects the expanded extracellular fluid (ECF) volume (lowers the uric acid level) resulting in increased urinary Na excretion (elevated urine Na).

It is important to emphasize that ADH levels are also elevated in most other types of hyponatremias (e.g., hypervolemic or hypovolemic), in response to the decreased effective arterial blood volume (EABV) leading to decreased tissue perfusion. While hypovolemic patients have a true volume depletion, patients with edematous states also have a decreased EABV either from low cardiac output (heart failure) or from excessive vasodilation (cirrhosis). One should consider the elevated ADH levels in these states as “appropriate” as opposed to SIADH.

Diagnostic Criteria for SIADH

Clinical euvolemia is an important prerequisite for the diagnosis of SIADH, which can be easily established based on history (vomiting and/or diarrhea, use of diuretics) and physical examination (peripheral edema and/or ascites). It is important to rule out overt hypothyroidism and adrenal insufficiency before diagnosing the patient with SIADH. The main laboratory criteria are measured serum osmolality <275 mosmol/kg, serum uric acid less than 4 mg/dL, urine osmolality >100 mosmol/kg, and urine Na >40 mEq/L [4]. 

While the diagnostic cut-off for the urine osmolality in SIADH is >100 mosmol/kg, most SIADH patients with clinically significant hyponatremia have a urine osmolality in the range of 300-600 mosmol/kg. In patients with suspected SIADH, it is important to obtain urine studies as soon as possible (when not on IV fluids), which will help to guide the therapy with solutes. In some patients consuming a low Na diet, urine Na levels may appear in the range of 10-40 mEq/L despite meeting the other requirements of SIADH. In unclear cases with mild asymptomatic hyponatremia, repeating the urine chemistries after a 500-1000 ml 0.9% saline bolus can help to clarify the diagnosis of SIADH. Persistent hyponatremia despite the saline challenge with an elevated, relatively fixed urine osmolality is strongly suggestive of SIADH.

Hypouricemia in SIADH

SIADH can also be associated with hypouricemia (serum uric acid less than 4 mg/dL) which can be used as a supplemental criterion for SIADH in unclear cases. The occurrence of hypouricemia in SIADH patients is variable but is seen in most cases. SIADH patients have ECF volume which decreases the proximal tubular reabsorption of uric acid with resulting elevation (>10-12%) in FEUA and hypouricemia. ADH-induced activation of both the vasopressin 1 (V1) and vasopressin 2 (V2) receptors also plays an important role in hypouricemia [5]. Free water restriction in patients with SIADH typically improves both hyponatremia and hypouricemia.

Differential diagnosis of SIADH

Acute Dilutional Hyponatremia

Acute hyponatremia is suspected in symptomatic patients without baseline labs in conditions like water intoxication, transurethral resection of the prostate (TURP) syndrome, ecstasy use, and exercise-associated hyponatremia in athletes. Marathon runners and ecstasy users tend to drink large quantities of water and both conditions are also associated with inappropriate ADH secretion further contributing to hyponatremia. Monopolar electrosurgery procedures like transurethral resection of the prostate, bladder, and hysteroscopy may require large volume (around 30 L) irrigation with hypoosmotic fluids (glycine, sorbitol, and mannitol) to avoid thermal burns. Partial absorption of these hypotonic fluids via the venous circulation along with an elevated osmolar gap from the accumulation of substances can produce acute symptomatic hyponatremia [6]. The newer bipolar electrosurgery systems permit the use of isotonic saline which reduces the risk of hyponatremia [7]. Acute hyponatremia is a medical emergency since the rapid decrease in serum Na concentration by even a smaller margin (e.g., from 138 mEq/L to 128 mEq/L) can result in acute cerebral edema due to the shift of water from the hypotonic extracellular fluid into the brain. This can cause seizures, cerebral herniation, and death.

Elevated Serum Osmolality With Hyponatremia (Hypertonic Hyponatremia)

In some cases of euvolemic hyponatremia, measured serum osmolality may appear elevated in the presence of hyperglycemia, elevated blood alcohol levels, and administration of exogenous solutes like mannitol and intravenous immune globulins (suspended in mannitol). In hospitalized patients with diabetic ketoacidosis (DKA) and hyperosmolar hyperglycemic state (HHS), water moves out of the cells into the extracellular space thereby diluting all the electrolytes in the extracellular space including the Na. The approximate "corrected" Na for hyperglycemia varies from 1.6-2.4 mEq/L (average 2 mEq/L) for each 100 mg increase in glucose concentration [8]. It is important not to recalculate the anion gap after only correcting the serum Na, which will result in spurious anion gap elevation. If hyponatremia is persistent in patients with hypertonic hyponatremia, repeating the measured serum osmolality after the euglycemic state and after the metabolism of the substance (e.g., alcohol and mannitol) will help to further assess any underlying SIADH.

Normal Serum Osmolality with Hyponatremia (Pseudohyponatremia)

Pseudohyponatremia is a laboratory artifact that can occur in patients with extreme hypercholesterolemia (triglycerides, cholesterol, and lipoprotein X) or hyperproteinemia [9]. This only exists in vitro when “indirect” ion-selective electrodes are utilized by the laboratory analyzers. Arterial blood gas (ABG) machines use “direct” ion-selective electrodes, which are immune to such artifacts. Physicians should order blood gas analysis with electrolytes (ABG w/Lytes) where normal Na levels are revealed in most cases.

Hyponatremia in Patients with Advanced Renal Failure

The ability to excrete free water is significantly impaired in patients with advanced renal impairment (glomerular filtration rate <15 ml/min) and urine osmolality is also elevated (between 200 to 250 mosmol/kg) despite the appropriate suppression of ADH [10]. The resulting water retention can gradually lead to hyponatremia, but the simultaneous serum osmolality reduction is partially offset by the greater elevation in BUN.

Adrenal Insufficiency

Glucocorticoid deficiency from either primary or central causes can result in hyponatremia mediated by increased release of ADH with renal salt losses [11]. However, screening cortisol levels can be inappropriately low in hospitalized sick patients due to relative adrenal insufficiency and should be carefully interpreted. Hydrocortisone replacement therapy in true adrenal insufficiency can rapidly improve the hyponatremia and close monitoring is required to prevent overcorrection.

Hypothyroidism

Severe hypothyroidism can cause hyponatremia via uncertain mechanisms including failure to suppress the ADH levels [11]. Since thyroid function tests can be abnormal in many hospitalized patients (euthyroid sick syndrome), physicians should use clinical judgment in ordering further work. Patients with TSH elevations >20 mU/L are unlikely to be from euthyroid sick syndrome [12]. Hormone replacement therapy in patients with hypothyroidism will gradually resolve the hyponatremia.

Reset Osmostat

In some patients, the threshold plasma tonicity that regulates the release of ADH is abnormally low (e.g., decreases from 290 mosmol/kg to 260 mosmol/kg) and the pituitary gland regulates the serum Na around the new altered threshold (e.g., from 135 to 125 mEq/L). Causes of reset osmostat can be genetic and acquired (brain injury, chronic illness, malnutrition, tuberculosis, malignancy, dementia, and pregnancy) [13]. The hyponatremia in reset osmostat is often mild (rarely below 125 mEq/L) and is usually discovered in the screening blood tests.

The presence of a reset osmostat should be suspected in patients with milder forms of SIADH (serum Na >125 mEq/L) which is refractory to variations in Na and water intake. The water load test (performed under close supervision) can be used to differentiate reset osmostat from asymptomatic SIADH. After administering a water load (10 ml/kg oral or IV), patients with reset osmostat can generate dilute urine (urine osmolality below 100 mosmol/kg) when the serum osmolality falls below their lower-than-normal reset threshold for ADH. Patients with reset osmostat also have a normal (4-11%) fractional excretion of uric acid (FEUA). It is important to diagnose reset osmostat to avoid unnecessary investigations and treatment since it is not amenable to fluid restriction and salt supplementation.

Cerebral Salt Wasting (CSW)

CSW is a rare syndrome described in patients with intracranial pathology (e.g., subarachnoid hemorrhage, SAH) possibly due to the left ventricular release of B-type natriuretic peptide (BNP) and/or diminished central sympathetic activity. CSW only accounts for <10% of cases and SIADH still accounts for the vast majority of cases of hyponatremia associated with SAH [14]. CSW mimics all of the findings in the SIADH (e.g., elevated urine Na and urine osmolality), and serum uric acid levels are also decreased due to urinary losses induced by elevated BNP levels. CSW can be differentiated from SIADH by subtle signs of volume depletion (e.g., orthostatic hypotension, decreased skin turgor, and increased BUN/creatinine ratio) and persistently elevated FEUA of >10% despite correction of hyponatremia [15]. In patients with CSW aggressive fluid restriction may promote cerebral vasospasm and symptomatic patients should be treated with 3% saline to ensure a prompt increase in the serum Na concentration. Salt tablets or fludrocortisone can be administered once the patients can take oral medications. CSW tends to be of transient duration and resolution usually occurs within three to four weeks.

The term “renal salt wasting” is used to describe a similar presentation of hyponatremia that occurs without any cerebral disease [16]. Some patients with traumatic brain injury can have both hyponatremia (SIADH/CSW) and elevation in intracranial pressure (ICP). Symptomatic patients may benefit from the use of 23.4% hypertonic saline (30 ml IV push over 10 min) administered via central line, which results in a significant decrease in ICP within one hour of use. 

Thiazide-Induced Hyponatremia

Thiazides can rarely cause hyponatremia (which can be severe) soon after initiation of therapy and also at any time during long-term use. The mechanisms of thiazide-induced hyponatremia are complex, and most patients are clinically euvolemic on presentation [17]. These patients not only tend to increase daily water intake but also manifest an impairment in water excretion from enhanced ADH release. Hypouricemia is known to occur in SIADH-related cases of thiazide-induced hyponatremia [18]. Discontinuation of thiazides will result in marked improvement of the serum Na over days to two weeks in most cases. Thiazides should be discontinued after an episode of hyponatremia since rechallenge in most cases will result in recurrence [19].

Primary Polydipsia

Patients who drink large quantities of water (primary polydipsia) can be differentiated from SIADH by the history of water intake, polyuria (>3L/day), and persistently dilute urine with a urine osmolality less than 100 mosmol/kg. Compulsive water drinking is most often seen in patients with psychiatric illnesses (~6% incidence), including those taking antipsychotics (e.g., phenothiazine) that can lead to the sensation of a dry mouth. A downward resetting of the osmostat regulating ADH release is also observed in some patients with psychogenic polydipsia [20]. Primary polydipsia can also be induced by hypothalamic infiltrative diseases (e.g., sarcoidosis) that can directly affect the thirst center. Normal subjects can excrete more than 500ml of urine per hour, a response that is mediated by the suppression of ADH secretion and intact kidney function. Since the excess water is readily excreted, primary polydipsia should not lead to severe hyponatremia without a massive increase in daily water intake. Concurrent SIADH is assumed to have a contributory role in patients with urine osmolality in the range of 100-150 mosmol/L and treated accordingly by increasing oral solute intake.

Low Dietary Solute Intake (Beer Potomania and Tea & Toast Syndrome)

Ingestion of a normal diet results in the generation and excretion of 600-900 mosmol of solute per day containing primarily Na and potassium salts along with urea. Patients who drink large quantities (>4 L/day) of beer (beer potomania) have decreased daily solute excretion (beer contains little Na 1-2 mEq/L, potassium, or protein) along with a suppressed urea production from carbohydrate load [21]. Other malnourished patients on low protein, high water-intake diets (e.g., tea and toast syndrome) may also have a marked reduction in water excretory capacity that is directly mediated by poor dietary solute intake. The symptoms of this hypo-osmolality syndrome include fatigue, dizziness, and muscular weakness, which can be easily corrected by 0.9% saline administration (osmolality 308 mEq/L).

Treatment of SIADH general principles

Treatment of SIADH should be individualized based on the clinical presentation with a common focus on daily water restriction and increased solute intake (oral urea powder, salt tablets) along with loop diuretics and vasopressin antagonists in some cases (Table 2). Excess water accompanies the solutes when they are excreted into the urine, as long as the administered solution/substance osmolality exceeds the urine osmolality of the patient [22]. In all patients with hyponatremia, the use of hypotonic fluids (e.g., D5W and 0.5% saline) should be avoided along with Ringer’s lactate, which has less Na concentration (130 mEq/L) compared to the 0.9% saline (154 mEq/L). 

A 0.9% Saline Infusion can Rapidly Correct Hyponatremia in SIADH

Physicians are generally too quick to administer 0.9% saline in the emergency departments and in many cases, it is done before the serum studies result. While 0.9% saline (154 mEq/L) is considered isotonic in normal subjects, it acts as a hypertonic solution in hyponatremic patients with a dual effect on the serum Na levels (can rapidly improve and/or worsen serum Na). During the infusion, 0.9% saline can quickly correct the serum Na levels and may induce osmotic demyelination in susceptible patients. In a Swedish study of 83 patients with osmotic demyelination, 93% of patients received 0.9% saline [3]. Concurrent administration of a loop diuretic can potentiate this effect of saline on Na overcorrection. It is recommended to avoid the use of 0.9% saline in patients with chronic SIADH (serum Na <125 mEq/L).

A 0.9% Saline Infusion can Worsen Hyponatremia in SIADH

The urine osmolality in most patients with significant SIADH is greater than 350 mosmol/kg, which is greater than the osmolality of 0.9% saline (308 mosmol/kg). A 0.9% saline infusion will temporarily increase the serum Na because it is hypertonic to the patient with a low serum Na. Since the patient is euvolemic, kidneys will excrete the entire infused Na into the urine, but only some of the free water accompanies the solute (due to the urine osmolality being higher than the 0.9% saline osmolality). 0.9% saline infusion (hypertonic to the patient with hyponatremia) may also result in transcellular water shifts as water moves from the intracellular to extracellular space to equalize the osmolality of the two fluid compartments. Retention of most of the daily consumed water (e.g., 1000ml) plus the translocated water (amount varies based on the serum Na level) in these patients may result in worsening of serum Na levels in the subsequent chemistries [23]. Hypertonic saline (osmolality 1026) is not associated with post-infusion hyponatremia due to the significant free water excretion into the urine, which is illustrated in the clinical example below. 

Administration of 1 L 0.9% saline (Na-154 mEq/L; osmolality 308 mosmol/L) to a hypothetic SIADH patient (with a serum Na 125, urine osmolality 500 mosmol/kg and consuming 1000 ml of free water per day) will have the below effect on the urinary water excretion. Kidneys will excrete the entire infused Na into the urine, but only around 600 ml of water (308/500 mosmol/kg = 616 ml) accompanies the solute due to the presence of elevated ADH levels. Retention of 400 ml of daily administered water in this example may lead to further worsening of hyponatremia (the effect of retained translocated water is not included in this example). When a loop diuretic is administered with 0.9% and hypertonic saline, it is assumed to lower the urine osmolality of the patient by 50% thereby resulting in additional free water excretion respectively (Table 2).

**Table 2:** Effect of solute administration on serum Na levels in SIADH Patient characteristics in the example: Daily 1000ml free water intake with a relatively fixed urine osmolality of 500 mosmol/Kg. Units of measurement: milliosmoles per kilogram of water (mosmol/kg). SIADH: syndrome of inappropriate antidiuretic hormone secretion; Na: sodium.

Substance osmolality/Urine osmolality	Net urine water loss vs gain	Comments
60 g urea (1000 mosmol/l)		
Expected urine output 1000/500 mosmol/kg = 2000 ml	2000 -1000 ml daily water consumed = 1000 ml net loss in total body water	Serum Na will gradually improve due to a 1000 ml daily loss in body water while consuming urea
0.9% Saline (308 mosmol/l)		
Expected urine output 308/500 mosmol/kg = 600 ml	600 -1000 ml daily water consumed = 400 ml net gain in total body water	A 0.9% saline use in this exam will worsen hyponatremia due to the retention of 400 ml of consumed water
0.9% saline + loop diuretic (Urine osmolality decreased to an assumed 250 mosmol/kg by loop diuretics)		
Expected urine output 308/250 mosmol/kg = 1500 ml	1230 -1000 ml daily water consumed = 230 ml net loss in total body water	Serum Na may improve due to 230 ml loss in bo230 ml a loop diuretic is administered. with 0.9% saline
3% saline (1026 mosmol/l)		
Expected urine output 1026/500 mosmol/kg = ~2050 ml	2050 -1000 ml daily water consumed = 1050 ml net loss in total body water	Serum Na will gradually improve due to a 1050 ml loss in body water
3% saline + loop diuretic		
Expected urine output 1026/250 mosmol/kg = ~4100 ml	4100 -1000 ml daily water consumed = 3100 ml net loss in total body water	Serum Na will rapidly improve due to a 3100 ml loss in body water. Such a combination should be avoided in clinical practice

Effect of Concurrent Potassium Repletion on Na Levels

Some patients with drug-induced hyponatremia (e.g., thiazides and crizotinib) may also present with severe hypokalemia. Potassium is an osmotically active substance and after administration of serum, Na levels may increase due to the movement of intracellular Na into the extracellular fluid. It is important to consider the risk of Na overcorrection in symptomatic SIADH patients who are receiving both potassium and hypertonic saline [24].

Treatment of SIADH with mild or no symptoms

Free Water Restriction

Water restriction to less than 1000 ml/day is the mainstay of initial therapy for all patients with hyponatremia. The associated negative water balance will gradually raise the serum Na concentration toward normal. Water restriction can be lowered to 800 ml/day in refractory cases, but patient compliance is difficult to adhere to for prolonged periods. Fluid restriction is not appropriate for patients who are at risk for cerebral vasospasm and infarction (e.g., hyponatremia associated with SAH). Fluid restriction alone results in a modest increase in serum Na level within three to four days, but several patients fail to reach the goal Na level of >130 mEq/L without additional therapies [25].

In addition to the free water restriction, there are several other available treatment modalities to improve serum Na concentration in patients with euvolemic hyponatremia (Table 3).

**Table 3:** Treatment of SIADH SIADH: syndrome of inappropriate antidiuretic hormone secretion. Units of measurement: milliosmoles per kilogram of water (mosmol/kg).

Name	Dose	Notes
Urea powder 30 g (500 milliosmoles)	30-60 g/day in divided doses	Urea can be used to treat chronic SIADH with few adverse effects. Urea has a relatively cheaper price for inpatient use (8 doses around $15). Urea has a bitter taste (mix with sweeteners or orange juice).
Salt tablets 9	9 g/d in divided doses (e.g., 3 grams thrice a day)	Salt tablets 9-gram total daily dose equals the amount of Na in 0.9% saline. Salt tablets are better suited to treat young patients with SIADH with no comorbid conditions and should be used with caution in patients with hypertension.
Loop diuretics	Torsemide 20mg/daily or furosemide 20mg bid	Loop diuretics can be used to enhance the efficacy of oral salt tablets in selective patients prone to developing hypertension or edema. Renal dysfunction occurs commonly after prolonged use in euvolemic patients. Loop diuretics should be used with caution along with saline (0.9 or 3%), which can lead to rapid correction of serum Na.
0.9% saline (Na- 154 mEq/L; osmolality 308 mosmol/kg)	500ml to 1 l bolus x 1 then repeat serum Na	0.9% saline challenge can be administered in patients with mild hyponatremia (Na >125 mEq/L) of unclear etiology (e.g., urine Na levels <20). Avoid use in moderate to severe SIADH (Na <125 mEq/L) as serum Na levels can rapidly correct during the infusion and may also worsen after stopping the infusion.
3% saline (Na- 513 mEq/L Osmolality 1026 mosmol/kg)	50-100 ml bolus repeat x 2	Only use in patients with severe neurologic symptoms resulting from either acute or chronic hyponatremia. Hypertonic saline can be safely infused via peripheral vein.
Direct vasopressin receptor antagonists	Tolvaptan 7.5-15mg initial dose	Vaptans should always be initiated using the lowest possible dose. Vaptan use can stimulate thirst and they should be avoided in cirrhosis. Vaptans remain very expensive for routine use (around $300 per tablet).
Indirect vasopressin receptor antagonists	Demeclocycline 300 to 600 mg twice a day	Effect of demeclocycline may take up to one week to become apparent. It can cause nausea, vomiting, photosensitivity, and nephrotoxicity. Demeclocycline is expensive (around $300 for 60 tablets) for long term use.

Increasing the Urinary Solute Excretion with Urea

Urea administration (30 g once or twice a day) will improve the serum Na concentration by increasing the excretion of electrolyte-free water [26, 27]. For example, 30 g of urea rapidly distributes into the TBW resulting in a 20-mosmol increase in serum osmolality (without any expansion of the ECF volume), and excretes within 12 hours into the urine resulting in a loss of around 1 L of electrolyte-free water due to its diuretic properties. Urea is relatively inexpensive, devoid of adverse effects for long-term use and the bitter taste can be overcome by mixing in orange juice.

Increasing the Urinary Solute Excretion with Salt Tablets

Calculating the dose of oral salt tablets uses the same principles as intravenous saline. For example, the administration of 9 g of oral salt (e.g., 3 g thrice a day) provides a similar quantity of Na as 1 L of isotonic saline. Since the salt tablets are absorbed gradually compared to the IV 0.9% saline infusion, rapid serum Na fluctuations and water translocation effects are rare. Oral salt tablets should be used with caution in SIADH patients with comorbid conditions (e.g., hypertension and left ventricular dysfunction) and should be discontinued if edema develops during therapy.

Effect of Loop Diuretics in SIADH

The effect of salt administration can be enhanced when given with a loop diuretic that lowers the urine osmolality and increases water excretion [28]. By decreasing the Na chloride reabsorption in the medullary aspect of the loop of Henle, loop diuretics directly interfere with the countercurrent concentrating mechanism and induce a state of ADH resistance. In clinical practice, loop diuretic administration results in a lowering of urine osmolality by approximately 50% (e.g., 500 mosmol/L decreases to 250 mosmol/L) in patients with SIADH. It is important to understand the risk of serum Na overcorrection when loop diuretics are concurrently used with saline (0.9 or 3%) administration. Many patients require potassium chloride supplementation while on loop diuretics and renal failure occurs frequently in euvolemic patients precluding long-term use.

Use of Vasopressin Receptor Antagonists

Vaptans are direct ADH antagonists that produce selective water diuresis (aquaresis) without affecting Na and potassium excretion and are well-studied in the treatment of hyponatremia associated with both SIADH and fluid overload states. Agents like tolvaptan are selective for the V2 receptor and others like conivaptan block both V1 and V2 receptors. Vaptans are associated with significant stimulation of thirst and patients should be advised to maintain the free water restriction. One should use the lowest starting dose in patients with SIADH due to the risk of massive water diuresis and possible overcorrection of serum Na levels [29]. While vaptans represent the most physiologic way to treat SIADH, they are too expensive to be used routinely in hospitalized patients.

Indirect ADH antagonists (e.g., demeclocycline) act on the collecting tubule cell to diminish its responsiveness to ADH, thereby increasing water excretion. They have delayed clinical effects and use can be associated with renal failure in some patients.

Despite a thorough workup (occult infections, malignancies) and screening for offending drugs, several patients may have an idiopathic form of SIADH. Once a safe serum Na level (128-130 mEq/L) is reached on solute therapy, patients can be discharged with close monitoring of serum Na with weekly blood draws till the stimulus to the ADH is resolved.

Treatment of symptomatic hyponatremia

Hypertonic saline is indicated in the treatment of all patients with moderate to severe symptoms of acute or chronic hyponatremia (e.g., seizures, obtundation, coma). Hypertonic saline can be safely administered via the peripheral vein to rapidly increase the serum Na by 4 mEq/L over a few hours, which relieves the symptoms in most cases. Maximum daily Na correction should not exceed 8 mEq/L per 24 hours in any patient with acute or chronic hyponatremia [30].

Avoid the use of Predictive Formulae

Several predictive formulae have been proposed to estimate the direct effect of a given saline solution on the serum Na concentration. There are several variables to consider in hospitalized patients that may make these estimates inaccurate [31]. In some cases, there is a quick reversal to the ADH stimulus (e.g., postoperative pain and nausea) resulting in massive diuresis. One should also account for the daily insensible water losses (500 ml/d) and the effect of any other osmotically active substances (e.g., potassium) that may contribute to the elevation in serum Na. Frequent monitoring of the serum Na levels (e.g., every one to two hours till Na levels are above 120 mEq/L) remains the best way to prevent overcorrection of SIADH.

Dosing of Hypertonic Saline

Urine osmolality in severe SIADH patients is typically in the range of 600 mosmol/Kg and infusion of 3% saline (Na-513; osmolality-1027) will result in correction of serum Na levels. There is unlikely a post-infusion reduction in serum Na levels (as seen with 0.9% saline) due to the extensive water losses into the urine. Due to the high risk of overcorrection, 3% saline is only given in small volume boluses as an initial 100 ml (1.5 to 2 ml/kg) bolus, repeated as needed (every 10 minutes x 2) in patients with persistent symptoms. After a total dose of 300 ml, no further hypertonic saline should be administered till the serum Na is remeasured [32, 33].

Monitoring Urine Output and Treatment of Excess Serum Na Correction

Since most cases of ODS have severe and irreversible neurologic consequences, prevention is essential. It is not possible to accurately estimate the effect of saline on the serum Na as some patients will correct it too rapidly due to the massive aquaresis. In SIADH patients receiving hypertonic saline or vaptans, the urine volume should also be monitored along with frequent serum Na levels. In patients prone to developing ODS (e.g., alcoholics and symptomatic hyponatremia with level <120 mEq/L) preventative administration of desmopressin (DDAVP 1-2 µg IV/SQ every six to eight hours) is preferred along with the hypertonic saline [34]. If abrupt aquaresis (e.g., clear dilute urine exceeding 150ml per hour) ensues during therapy, the urinary free water losses can be replaced with D5W while adjusting the doses of desmopressin and hypertonic saline. DDAVP is known to dramatically decrease the rate of serum Na overcorrection and higher doses are required in patients who are being treated with vaptans [35].

## Conclusions

After the initial assessment of the hospitalized euvolemic patient with hyponatremia, the diagnosis of SIADH is relatively straightforward with a few simple criteria. SIADH patients without any baseline electrolytes collected in the last 48 hours should be assumed to have a chronic form, which makes them highly prone to osmotic demyelination. Checking urine osmolality is vital in the management of SIADH to ensure that the osmolality of the administered solute is exceeding the urine osmolality. Asymptomatic SIADH patients should be treated with free water restriction and increasing daily oral solute intake (urea or salt tablets). Oral urea administration is underutilized despite being widely available and affordable. It is important to avoid indiscriminate use of 0.9% saline in patients with SIADH, which can not only cause osmotic demyelination from rapid Na correction but also can result in post-infusion worsening of serum Na levels. Hypertonic (3%) saline use should be restricted to patients with severe neurologic symptoms till the serum Na levels are gradually (maximum daily correction <8 mEq/L) improved to a level of >120 mEq/L. The use of adjunctive parenteral desmopressin can decrease serum sodium overcorrection while being treated with hypertonic saline.

In patients with intracranial pathology (e.g., recent intracranial surgery or subarachnoid hemorrhage), SIADH remains the most common cause of hyponatremia and CSW occurs only in a small number of cases. Irrespective of etiology, all patients with active intracranial pathology and symptomatic hyponatremia should have a prompt increase in the serum sodium concentration with hypertonic saline which will correct both CSW and SIADH.
